# LNK promotes granulosa cell apoptosis in PCOS via negatively regulating insulin-stimulated AKT-FOXO3 pathway

**DOI:** 10.18632/aging.202421

**Published:** 2021-01-20

**Authors:** Min Tan, Yanxiang Cheng, Xiaozhu Zhong, Dongyong Yang, Sushi Jiang, Yang Ye, Miao Ding, Guijun Guan, Dongzi Yang, Xiaomiao Zhao

**Affiliations:** 1 Department of Obstetrics and Gynecology, Sun Yat-sen Memorial Hospital, Sun Yat-sen University, Guangzhou 510120, China; 2 Department of Obstetrics and Gynecology, Renmin Hospital of Wuhan University, Wuhan 430060, Hubei, China; 3 Shanghai Collaborative Innovation for Aquatic Animal Genetics and Breeding, College of Fisheries and Life Sciences, Shanghai Ocean University, Shanghai 201306, China

**Keywords:** polycystic ovary syndrome, insulin resistance, LNK, FOXO3, apoptosis

## Abstract

Background: Polycystic ovary syndrome (PCOS), which is often accompanied by insulin resistance, is closely related to increased apoptosis of ovarian granulosa cells. LNK is an important regulator of the insulin signaling pathway. When insulin binds to the receptor, the PI3K/AKT/FOXO signaling pathway is activated, and FOXO translocates from the nucleus to the cytoplasm, thereby inhibiting the expression of pro-apoptotic genes.

Methods: Granulosa cells were collected from PCOS patients to investigate the relationship between LNK, cell apoptosis and insulin resistance. KGN cells underwent LNK overexpression/silence and insulin stimulation. The AKT/FOXO3 pathway was studied by western blot and immunofluorescence. *LNK* knockout mice were used to investigate the effect of LNK on the pathogenesis of PCOS.

Results: The level of LNK was higher in PCOS group than control group. LNK was positively correlated with granulosa cell apoptosis and insulin resistance, and negatively correlated with oocyte maturation rate. LNK overexpression in KGN cells inhibited insulin-induced AKT/FOXO3 signaling pathway, causing nucleus translocation of FOXO3 and promoting granulosa cell apoptosis. *LNK* knockout partially restored estrous cycle and improved glucose metabolism in PCOS mice.

Conclusions: LNK was closely related to insulin resistance and apoptosis of granulosa cells via the AKT/FOXO3 pathway. *LNK* knockout partially restored estrous cycle and improved glucose metabolism in PCOS mice, suggesting LNK might become a potential biological target for the clinical treatment of PCOS.

## INTRODUCTION

Polycystic ovary syndrome (PCOS) is a common endocrine and metabolic disease that affects 7-9% women of reproductive age [1]. PCOS is usually characterized by ovulation dysfunction, androgen excess and polycystic ovaries observed by ultrasound. PCOS patients are often accompanied by overweight, insulin resistance (IR) and glucose metabolism disorders [2]. Studies show that the high insulin level in PCOS promotes granulosa cell (GC) apoptosis [3, 4] which leads to follicular developmental disorders. However, in PCOS patients with IR, the mechanism by which high insulin promotes GC apoptosis has not been fully elucidated.

LNK (SH2B3) belongs to the Src homology 2B (SH2B) family which contains SH2 and PH domains. The family consists of intracellular adaptor proteins which regulate various pathways. LNK is considered as an important regulator of insulin signaling pathway, and plays an important part in glucose homeostasis as well as reproduction [5]. Our previous studies showed that levels of LNK were elevated in ovaries of insulin resistant PCOS patients compared with the non-PCOS group, and that LNK co-localized with insulin receptor [6]. In addition, overexpression of LNK in the human ovarian granulosa cell line (KGN) inhibited insulin-induced AKT activation [6].

The forkhead box O (FOXO) family plays a vital role downstream of the insulin signaling pathway [7] and actively participates in a variety of cellular and physiological processes including cell proliferation, apoptosis and the regulation of cell cycle [8, 9]. FOXO's subcellular localization and function are controlled by post-transcriptional modifications such as acetylation and phosphorylation [10], and insulin plays an important role in the process [11]. In the nucleus, FOXO triggers apoptosis by inducing the transcription of pro-apoptotic genes such as FasL, and thus actively participates in the process of apoptosis. When insulin or other growth factors are present, FOXO proteins are relocated from the nucleus to the cytoplasm [12]. PI3K/AKT is one of the most important pathways in regulating FOXO function in different types of organisms [11]. When insulin or other growth factors bind to their receptors, the PI3K/AKT pathway is activated, and activated AKT phosphorylates FOXO, thereby negatively regulating the nuclear localization of FOXO [9]. Studies suggest that insulin can activate the PI3K/AKT pathway in granulosa cells (GCs) [13]. Moreover, GC apoptosis is increased in PCOS patients with high insulin levels [3]. Therefore, we speculate that there is a complex mechanism for regulating granulosa cell apoptosis in PCOS patients with IR.

Therefore, we hypothesize that LNK negatively regulates insulin-activated AKT/FOXO3 pathway and promotes granulosa cell apoptosis and dysfunction, therefore affecting oocyte maturation and thus participates in the etiology of ovulation disorder in polycystic ovary syndrome.

In our study, we demonstrate that LNK expression is increased in PCOS patients, and the higher LNK level in KGN can inhibit the AKT/FOXO3 pathway, thereby inducing apoptosis of the granulosa cells. LNK knockout can partially restore estrous cycle and improve glucose metabolism in PCOS mice.

## RESULTS

### LNK was positively correlated with the severity of insulin resistance and granulosa cell apoptosis, and negatively correlated with oocyte maturation rate

**Figure 1:**
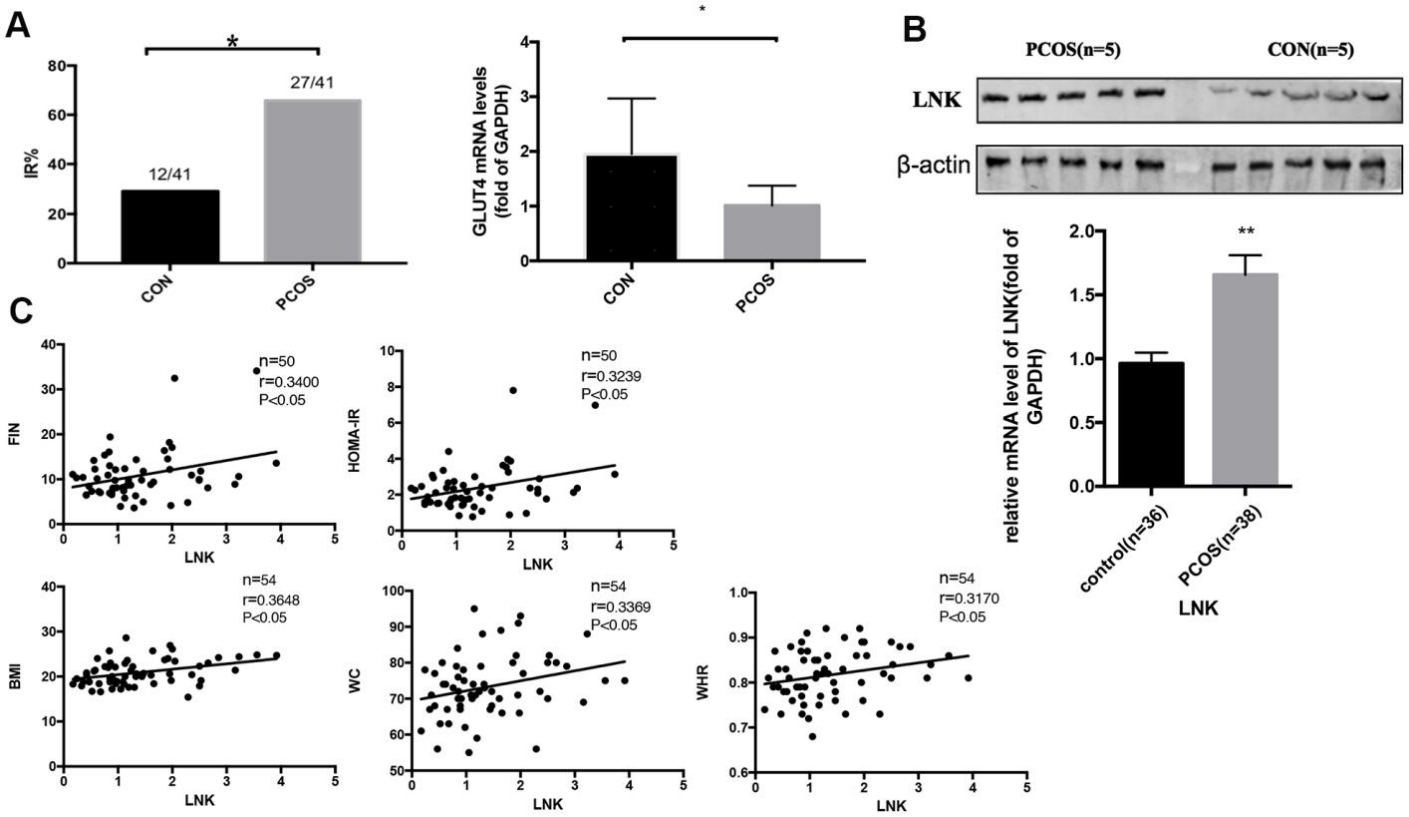
**The incidence of insulin resistance in PCOS patients and control group, and the expression of LNK in granulosa cells from PCOS and control group.** (**A**) PCOS patients had a higher incidence of insulin resistance. GLUT4 mRNA level was increased in PCOS group. Results are mean±SD. *p < 0.05 vs con. (**B**) RT-PCR and western blot results showed higher levels of LNK mRNA and protein expression in granulosa cells in PCOS group compared with control group. Results are mean±SEM. *p < 0.05, **p < 0.01 vs. data of control group. (**C**) Pearson correlation analysis showed a positive correlation between LNK (relative mRNA level, fold of GAPDH) and clinical insulin resistance parameters in the total population.

We obtained metabolic profiles of the included subjects and found that the incidence of IR in PCOS patients was higher. 27 and 12 women were diagnosed as IR in PCOS and control group, respectively. Table 1 shows the characteristics of patients involved in this research. RT-PCR indicated that GLUT4 mRNA expression in luteinized GCs from the PCOS group was lower than that in the control group (Figure 1A), indicating that there was a certain degree of glucose metabolic disorder. Meanwhile, we detected the level of LNK in luteinized granulosa cells in different groups, and found that compared with the control patients, the level of LNK in PCOS patients was increased (Figure 1B). Pearson correlation analysis revealed that the level of LNK was positively correlated with the severity of insulin resistance from the total population (Figure 1C). Respective correlation analysis on PCOS and control group can be found in Supplementary Tables 3, 4 and Supplementary Figure 2. Generally speaking, the r values were higher when correlation analysis was done in PCOS group.

**Table 1:** Clinical characteristics of PCOS and control group.

	**Control (n=41)**	**PCOS (n=41)**	***p* value**
Age (years)	31.40±3.60	30.00±3.70	0.12
BMI	20.22±3.70	21.60±3.40	0.05
WC (cm)	71.70±7.00	76.10±9.70	<0.05
Fasting Glucose (mmol/L)	5.0±0.30	5.10±0.50	0.27
WHR	0.80±0.06	0.83±0.06	<0.05
Fasting Insulin (mU/L)	10.40±3.60	14.20±10.20	<0.05
HOMA-IR	2.10±0.80	3.30±2.60	<0.05
AMH (ng/mL)	3.20±2.00	10.10±5.50	<0.05
TT (nmol/L)	1.20±0.50	1.50±0.60	0.61
FSH (IU/L)	7.00±1.90	6.60±1.60	0.42
LH (IU/L)	4.70±1.90	7.80±5.10	<0.01

BMI, body mass index; WC, waist circumference; TT, total testosterone; FSH (IU/L), follicular stimulating hormone; LH (IU/L), Luteinizing hormone; AMH (ng/ml), Anti Mullerian Hormone; TT, Total testosterone.

**Figure 2:**
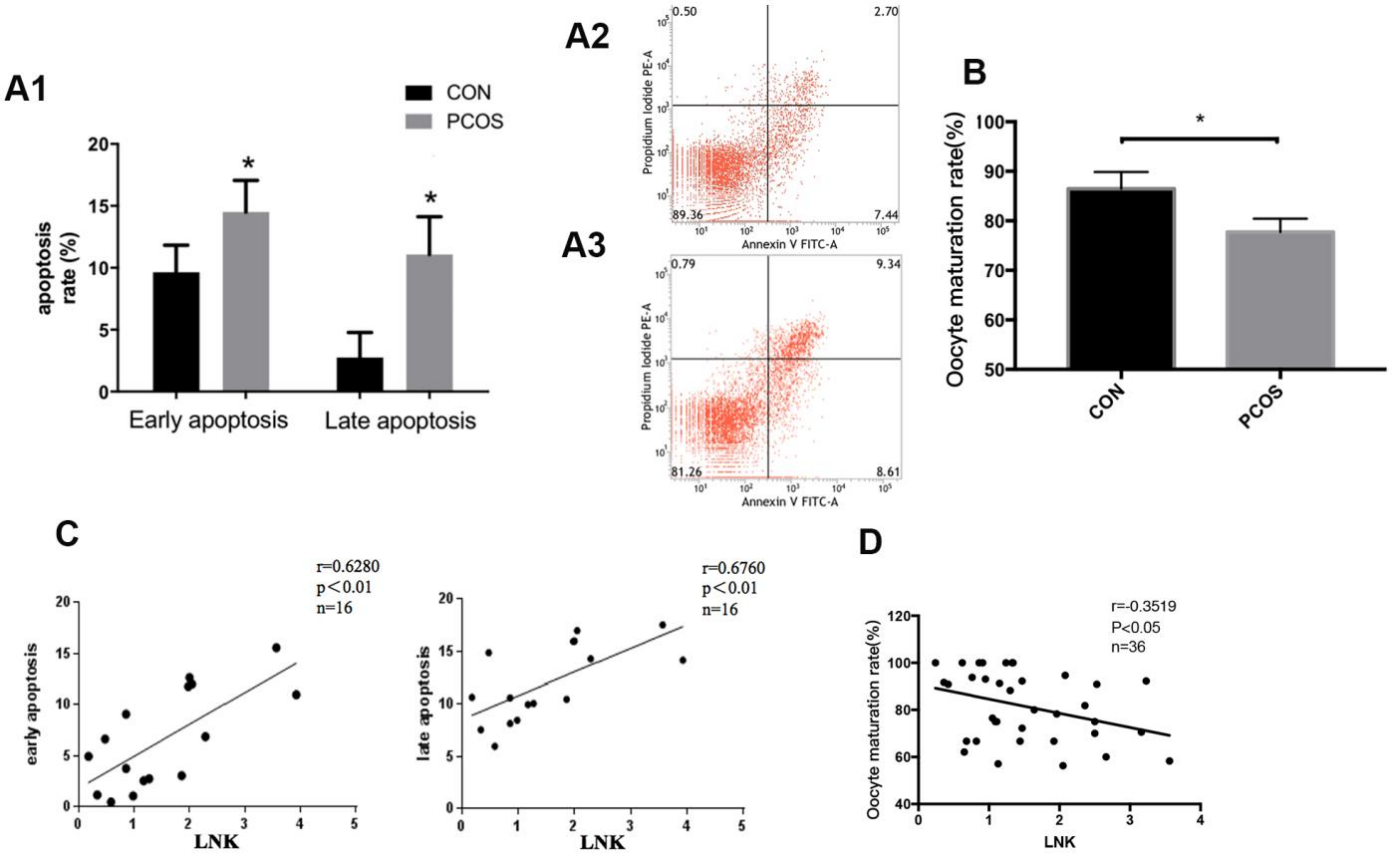
**Granulosa cell apoptosis and oocyte maturation rate in PCOS and control group.** (**A1**) Comparison of early and late apoptosis rate of granulosa cells in PCOS and control group by Annexin V FITC-A flow cytometry analysis. Results are mean±SD.*p < 0.05 vs. data of control group. (**A2, A3**) Examples of the Annexin V FITC-A flow cytometry analysis results of control group (**A2**) and PCOS group (**A3**). n=8 for each group. (**B**) PCOS patients had a lower oocyte maturation rate (number of mature oocytes/total retrieved oocytes) than control group. Results are mean±SEM. *p < 0.05 vs. data of control group. (**C**) Pearson correlation analysis showed a positive correlation between LNK (relative mRNA level, fold of GAPDH) and apoptosis. (**D**) LNK (relative mRNA level, fold of GAPDH) was negatively correlated with oocyte maturation rate.

In order to further investigate the changes in reproductive function of patients with PCOS, we examined the apoptosis level of luteinized GCs and oocyte maturation rate in both groups. Results showed that the apoptosis level of luteinized GCs from PCOS patients was higher compared with the control group (Figure 2A), and the maturation rate of oocytes in PCOS patients was significantly lower (Figure 2B). Interestingly, Pearson correlation analysis revealed that LNK expression was positively correlated with granulosa cell apoptosis rate, and was negatively correlated with oocyte maturation rate (Figure 2C, 2D).

These results indicate that LNK is elevated in GCs of PCOS patients, and may be an important regulator in insulin resistance, granulosa cell dysfunction and follicular development.

### FOXO3 was increased in granulosa cells of PCOS patients and positively correlated with IR, LNK, and apoptosis

**Figure 3:**
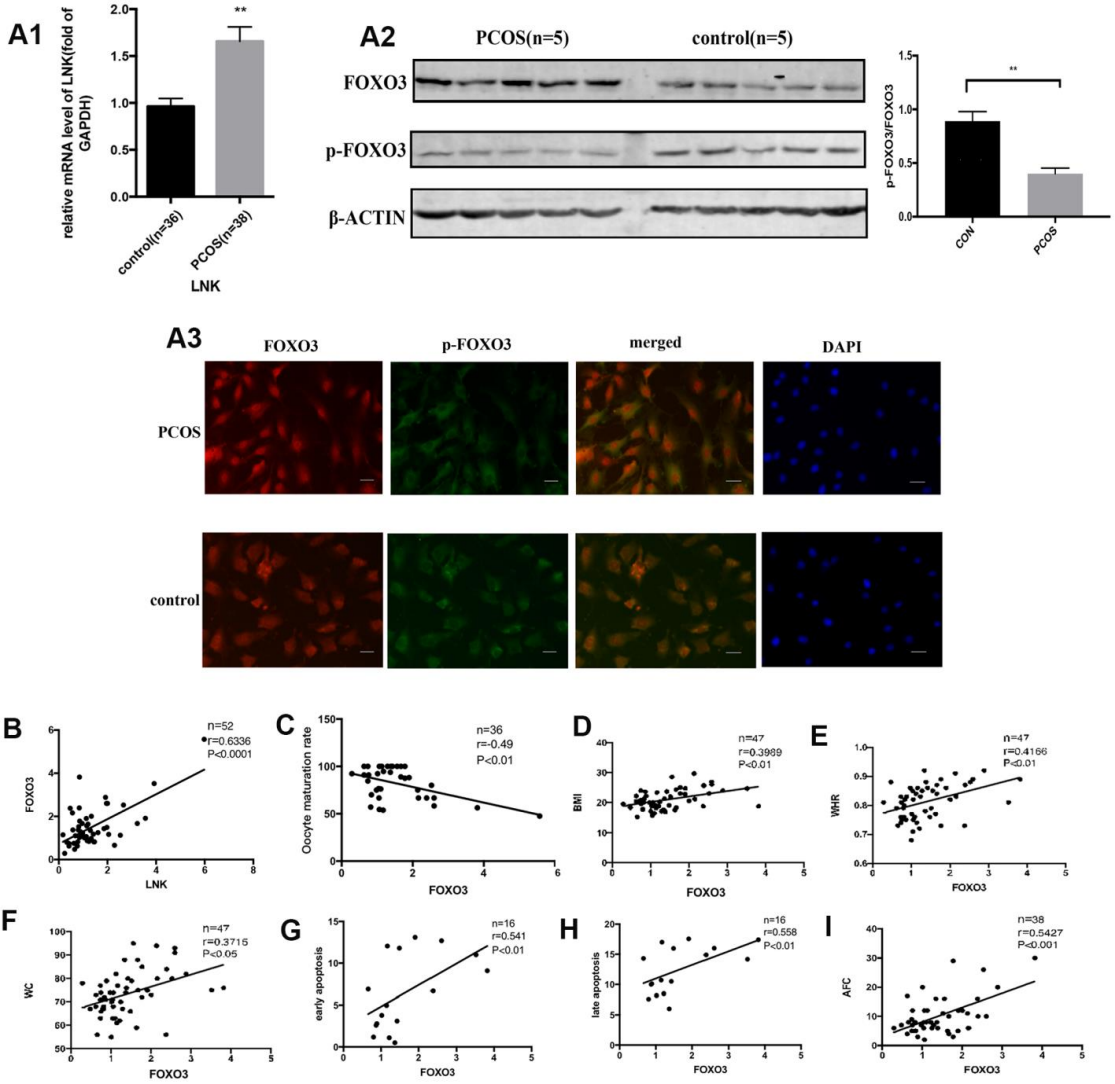
**The expression of FOXO3 in granulosa cells from PCOS and control group, and the relationship between FOXO3 and LNK, apoptosis, IR parameters, and oocyte maturation rate.** (**A1**) RT-PCR results showed a higher mRNA level of FOXO3 in granulosa cells in PCOS group compared with control group. Results are mean±SEM. (**A2**) Western blot analysis showed that p-FOXO3/FOXO3 level was decreased in PCOS patients compared with control group. Results are mean±SD.*p< 0.05, **p < 0.01 vs. data of control group. (**A3**) Immunofluorescence staining of granulosa cells showed that in PCOS group, FOXO3 expression was increased in the nucleus compared with control group. Scale bar, 20μm. (**B**) Pearson correlation analysis showed a positive correlation between LNK (relative mRNA level, fold of GAPDH) and FOXO3 (relative mRNA level, fold of GAPDH). (**C**) Pearson correlation analysis showed a negative correlation between oocyte maturation rate(%) and FOXO3 (relative mRNA level, fold of GAPDH). (**D**–**F**) Pearson correlation analysis showed a positive correlation between FOXO3 (relative mRNA level, fold of GAPDH) and clinical insulin resistance parameters. (**G**, **H**) Pearson correlation analysis showed a positive correlation between FOXO3 (relative mRNA level, fold of GAPDH) and apoptosis. (**I**) Pearson correlation analysis showed a positive correlation between FOXO3 (relative mRNA level, fold of GAPDH) and antral follicle counts.

The FOXO transcription factor family participates in many cellular processes, such as apoptosis and proliferation, and plays an important role downstream of the insulin and insulin-like growth factor receptors [14]. We detected the levels of FOXO3 in GCs of PCOS and control group. Results showed that FOXO3 mRNA expression was significantly elevated in PCOS group (Figure 3A1). Western blot analysis showed that p-FOXO3/FOXO3 level was lower in PCOS group (Figure 3A2). Immunofluorescence staining of GCs showed that in PCOS group, FOXO3 expression was elevated in the nucleus compared with control group (Figure 3A3). Then we investigated the relationship between FOXO3 and apoptosis, LNK, IR, and oocyte maturation rate with Pearson correlation analysis. The results showed that FOXO3 mRNA expression was positively correlated with LNK mRNA, cell apoptosis rate and IR-related parameters, and negatively correlated with oocyte maturation rate (Figure 3B–3I), indicating that FOXO3 dysregulation might play an important part in the apoptosis of GCs and follicular development disorder.

### Overexpression of LNK impaired insulin signaling and induced apoptosis of GCs

**Figure 4:**
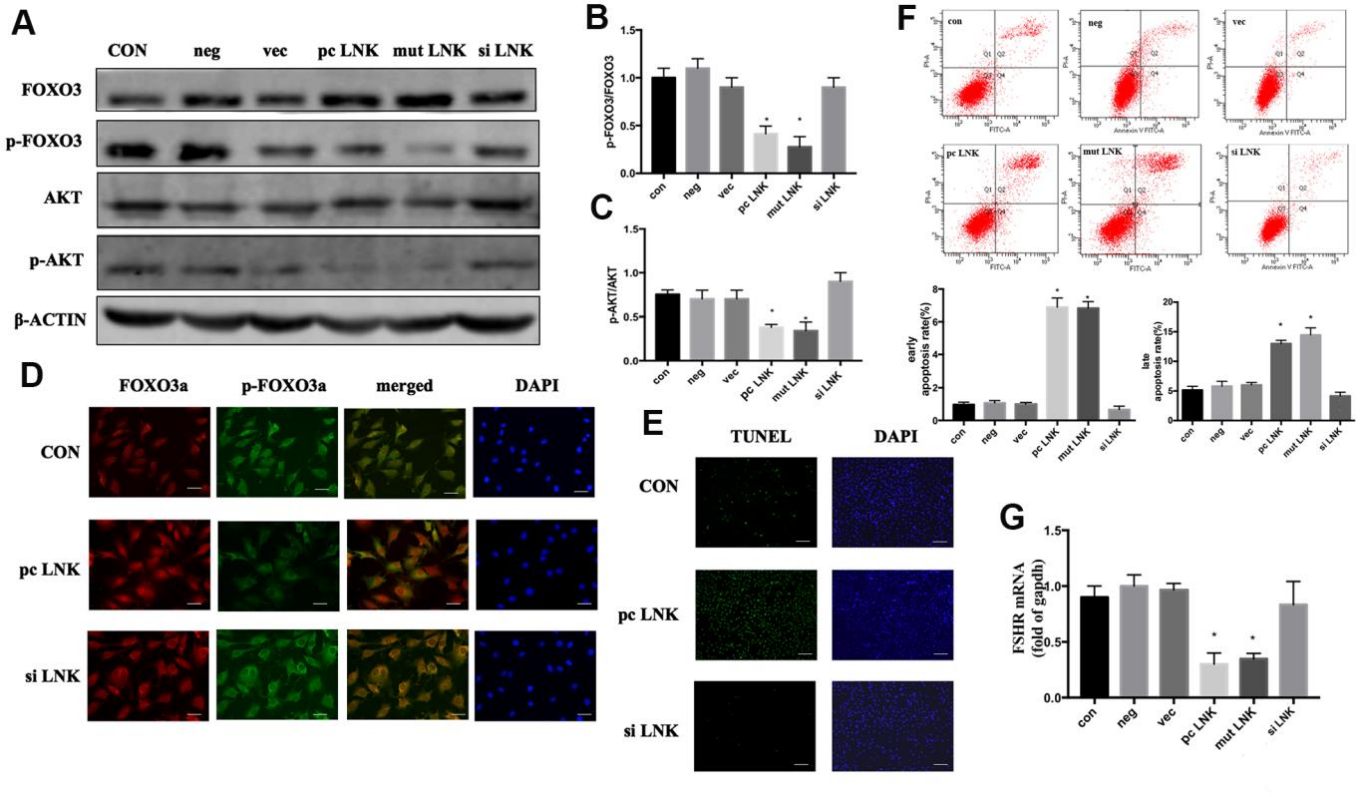
**Overexpression of LNK impairs insulin signaling and induces apoptosis of granulosa cells.** (**A**) Representative western blots of phosphorylated or total FOXO3 and AKT in LNK overexpression and silence granulosa cells. Ovarian granulosa cells were transfected for 48 hours and then starved for 12 hours with 1% FBS culture medium. After that, 100 nM insulin was added to the culture medium for 30 minutes, then cells were harvested for subsequent experiments. (**B**, **C**) Densitometry analysis of phosphorylation of AKT and FOXO3 in granulosa cells. The levels of phosphorylated AKT/AKT and phosphorylated FOXO3/FOXO3 were inhibited by overexpression of LNK in KGN cells. Results are mean±SD. *p < 0.05 vs. data of control group. (**D**) Immunofluorescence: pc LNK: FOXO3 dephosphorylated and its expression was increased in the nucleus. siLNK: FOXO3 was mainly expressed in the cytoplasm. Scale bar, 20μm. (**E**) Apoptosis detection of KGN was analyzed by TUNEL staining. Scale bar, 200μm. (**F**) Comparison of early and late apoptosis rate of KGN cells by Annexin V FITC-A flow cytometry analysis. Values are shown as means±SD, n=3, *p<0.05 vs. Con. (**G**) Comparison of FSHR. The KGN cells were transfected for 48 hours, then cells were harvested for RNA extraction. Values are shown as means ± SD, n=3, *p<0.05 vs. Con.

Patients with PCOS are often accompanied by IR [15]. When insulin or other growth factors are present, the AKT/FOXO3 pathway is activated, and FOXO3 is transferred from the nucleus to the cytoplasm, thereby inhibiting the expression of pro-apoptotic factors [16]. Otherwise FOXO3 is translocated to the nucleus and induces apoptosis. In order to explore the relationship and interaction mechanism between LNK, FOXO3 and granulosa cell apoptosis, KGN cells were transfected with LNK pcDNA3.1 (pc LNK), LNK mutant pcDNA3.1 (mut LNK), LNK siRNA (si LNK), empty pcDNA3.1 vector (vec) or negative control RNA (neg). After treated with insulin, the levels of AKT and FOXO3, the subcellular localization of FOXO3, and the apoptosis level of KGN were detected. The results showed that LNK overexpression inhibited phosphorylation of AKT and FOXO3, promoted the nuclear localization of FOXO3 and the apoptotic level of KGN, and the opposite results were obtained when LNK was knocked down (Figure 4A–4G). Previous studies have revealed that FSHR level is closely related to PCOS [17, 18]. In this study, we found that LNK overexpression inhibited the expression of FSHR. (Figure 4G).

These results indicate that LNK overexpression can negatively regulate the insulin-induced AKT/FOXO3 pathway and promote KGN apoptosis, which may be closely related to the ovulation dysfunction in PCOS patients.

### LNK knockout partially restored estrous cycle and improved glucose metabolism in PCOS mice

**Figure 5:**
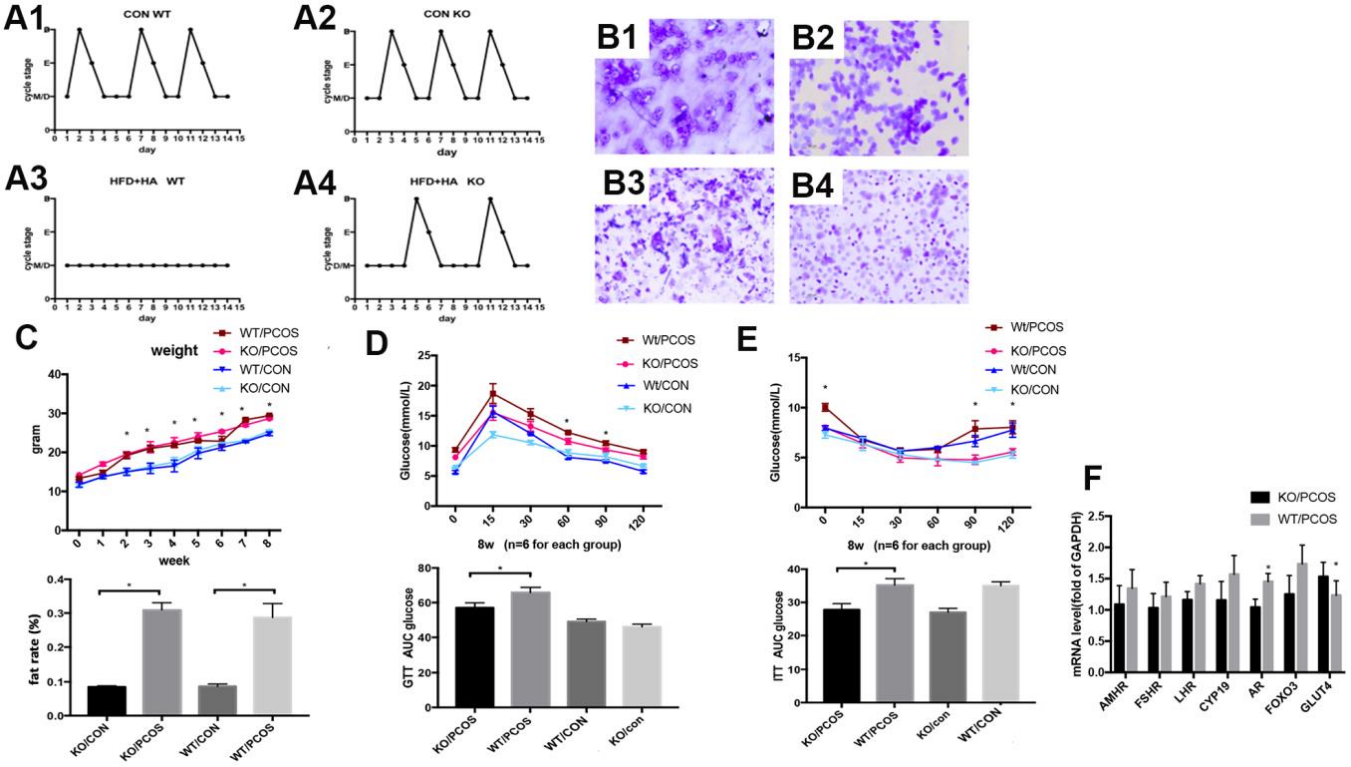
**LNK knockout partially restored estrous cycle and improved glucose metabolism in PCOS mice model.** (**A1**, **A2**) Control WT and KO mice had normal and regular estrous cycles. (**A3**) Irregular estrous cycle was found in WT mice treated with high fat diet + DHEA. (**A4**). Estrous cycle was partly restored in LNK KO mice treated with high fat diet + DHEA. (**B1**–**B4**) Different stages of mouse estrous cycle: pre-estrous (P), estrous (E), metestrus (M), and diestrus stage (D). (**C**–**E**) For each group (n=6), body weights (**C**) were measured in 0 to 8-week-old mice.*p< 0.05 PCOS vs control mice. Magnetic resonance imaging (MRI) analyses were shown to compare fat rate(%). GTT (**D**) and ITT (**E**) results. Data were analyzed with ANOVA followed by the Bonferroni multiple comparison post hoc test. *Statistical significance for WT/PCOS vs KO/PCOS. Area under curve was calculated for each group for GTT and ITT. Data were shown as means ± S.D. (**F**) The mRNA levels of GLUT4 and FSHR were detected in ovarian tissues of WT/PCOS and KO/PCOS. *p< 0.05.

In order to further explore the role of LNK in PCOS *in vivo*, wild-type PCOS mouse model (WT/PCOS) and PCOS mouse model with *LNK* gene knockout (KO/PCOS) were constructed. We monitored estrous cycle, body weight and glucose metabolism, and results showed that *LNK* KO could partially restore estrous cycle of PCOS mice (Figure 5A, 5B). The weight and fat rate of the PCOS mice (WT/PCOS and KO/PCOS) were higher compared with the control group (WT/CON and KO/CON) (Figure 5C). In addition, we did glucose tolerance test (GTT) as well as insulin tolerance test (ITT), and detected mRNA level of GLUT4. The results indicated that knocking out *LNK* significantly increased the level of GLUT4 and improved glucose metabolism in PCOS mice (Figure 5D–5F).

Above all, these results indicate that LNK is closely related to the estrous cycle and glucose metabolism of PCOS mice. The increased level of LNK in ovarian GCs of PCOS patients may be an important mechanism leading to ovulation dysfunction. Therefore, LNK may become a potential target for the clinical treatment of polycystic ovary syndrome.

## DISCUSSION

In this study, we found that LNK was closely related to insulin resistance and apoptosis of granulosa cells via the AKT/FOXO3 pathway. *LNK* knockout partially restored estrous cycle and improved glucose metabolism in PCOS model mice, suggesting LNK might become a potential biological target for the clinical treatment of PCOS.

Women with PCOS are often accompanied by insulin resistance, and high levels of insulin may be one of the causes of PCOS [19]. This study found that the incidence of IR in PCOS was significantly higher compared with the control group, and BMI, WHR, insulin level and HOMA-IR were significantly increased in PCOS group, which is consistent with previous studies. Studies show that hyperinsulinemia or excessive secretion of LH may cause abnormal response of granulosa cells to LH and thus impair follicular development [20–22]. Some studies imply that LNK is associated with the pathogenesis of human diseases including type 1 diabetes, hypertension, and cardiovascular diseases [23–25]. SH2B adaptor protein 3 (SH2B3), also named as LNK, is widely studied in malignant tumors [26, 27]. LNK is a negative signal-transduction regulator, which is widely involved in cytokine signaling and cell metabolism [28]. In previous studies, we have proposed that LNK is a significant factor in the development of IR in patients with PCOS and is closely related to the insulin signaling pathway in the ovary [29]. We have also found that LNK regulates glucose transport in adipose tissue through affecting the insulin-mediated IRS1/PI3K/AKT/AS160 pathway [29]. In the current study, we found that LNK level was significantly increased in granulosa cells of PCOS patients, and the expression was positively correlated with insulin resistance and GC apoptosis. These results indicate that the high level of LNK is closely related to granulosa cell dysfunction and insulin resistance in PCOS.

As an adaptor protein, LNK inhibits phosphorylated tyrosine proteins by recognizing and binding to them through its SH2 domain [28]. LNK is considered to be an important regulator of inflammation and insulin resistance in several tissues and organs [29]. In previous studies, we found that LNK and insulin receptors co-localized [13]. In this study, we discovered that elevated LNK impaired insulin-stimulated AKT and FOXO3 phosphorylation, thereby promoting nuclear localization of FOXO3 and leading to increased apoptosis of granulosa cells. In addition, knocking out LNK could effectively improve glucose metabolism and estrous cycle of PCOS mice, suggesting it might become a potential therapeutic target for PCOS.

In the current study, we found that GLUT4 mRNA level was elevated in *LNK* knockout PCOS mice. The mechanism of LNK-altered GLUT4 expression was further explored in our other work, in which similar results were found in HFD-induced insulin resistant mice [30].

Our previous DNA sequencing analysis of PCOS and control group found a rs78894077 gene polymorphism in exon 1, PH domain of *LNK*, and allele C was mutated to T (unpublished). By constructing the corresponding mutant LNK plasmid, we would like to preliminarily investigate the function of this polymorphism. Our current study showed that the cells transfected with mut LNK had similar results as pc LNK. We will further investigate into this polymorphism in our future study.

There are some limitations in this study. Molecular mechanism of the upstream regulation of LNK remains uncertain. Although our results show that LNK regulates FOXO3 function by affecting its phosphorylation status and subcellular location via the AKT pathway, the cause of FOXO3 up-regulation in granulosa cells from PCOS patients remains unclear. Some researchers reported that altered m6A modification was involved in the elevated level of FOXO3 mRNA expression in the luteinized granulosa cells from PCOS patients [31]. In addition, the effects of LNK on oocyte maturation and granulosa cell-oocyte interaction still need further study. Moreover, hyperandrogenism, another important feature of PCOS, is not fully explored in the current study. We will carry on related investigation in subsequent research work.

In conclusion, our study indicates that LNK expression is elevated in ovarian granulosa cells of patients with PCOS. LNK expression is closely related to insulin resistance, granulosa cell apoptosis, and oocyte maturation rate. LNK overexpression may promote granulosa cell apoptosis by inhibiting insulin-stimulated AKT/FOXO3 pathway. Compared with wild-type PCOS mice, glucose metabolism is improved, and the estrous cycle is more regular in PCOS mice with *LNK* knockout. This study suggests that LNK dysregulation may play a significant role in the pathogenesis of PCOS, and LNK might become a potential biological target for the clinical treatment of PCOS.

## MATERIALS AND METHODS

### Clinical samples

A total of 82 women aged between 20 and 40 years were enrolled in our study from 2016-1 to 2017-1. Among them were 41 PCOS patients diagnosed according to the Rotterdam criteria [32]. They were planned to receive *in vitro* fertilization and embryo transfer (IVF-ET) for anovulation (5 cases), oligo-ovulation (32 cases), or other reasons (4 cases) at the reproductive center, department of obstetrics and gynecology of Sun Yat-Sen Memorial Hospital. In addition, 41 non-PCOS women aged between 20 and 40 years with regular menstrual cycles, who were undergoing IVF-ET (long GnRH-a protocol) for tubal or male factor infertility, were enrolled as control group. The exclusion criteria were hyperprolactinemia (prolactin>25mg/L), thyroid dysfunction (hyperthyroidism or hypothyroidism), adrenal diseases, tumors that produce androgen or recent use of medications which might affect endocrine function (e.g., oral contraceptives). The hospital ethics committee approved the study, and we obtained written informed consents from all participants.

Physical examinations were performed on every participants. Height, weight, waist circumference (WC) and hip circumference were measured and body mass index (BMI) (kg/m^2^) and waist/hip ratio (WHR) was calculated. Participants were also evaluated for acne, acanthosis nigricans and terminal hair. To assess hirsutism, the modified Ferriman Gallwey (mFG) score [33] was applied. FSH, LH, total testosterone, fasting plasma glucose (FPG) (mmol/L) and fasting plasma insulin (FIN) (mU/L) levels were detected. Transvaginal ultrasound was performed to detect polycystic ovarian changes and calculate the number of antral follicles. Abnormal glucose metabolism was diagnosed according to the guidelines published by the American Diabetes Association (ADA) [34].

The homeostasis model assessment for insulin resistance (HOMA-IR), which is defined as (FPG (mmol/L) × FIN (μIU/mL)) / 22.5, is a widely-used index for the clinical evaluation of insulin resistance [35, 36]. We used 2.14 as the cut-off point for insulin resistance [37]. The participant who matched one of the following criteria was considered insulin resistant in this study: 1) delayed insulin peak (higher 2-hour insulin compared with 1-hour insulin in the oral glucose tolerance test), or pre-diabetes or type 2 diabetes mellitus defined by the American Diabetes Association or HOMA-IR ≥ 2.14; or 2) clinical observation of the presence of acanthosis nigricans as described in our previous study [6].

Oocyte maturation rate(%) = number of MII oocytes / total number of collected oocytes.

### Isolation of granulosa cells

All women received IVF treatment (GnRHa long protocol for ovarian stimulation). 36h after human chorionic gonadotropin was administered, oocyte retrieval was performed on the patient by aspirating follicular fluids. After picking up oocytes, the remaining follicle fluids were collected and centrifuged. Lymphocyte separation medium was used to isolate granulosa cells from the precipitate. Red blood cells were removed using lysis buffer. The cells were washed by centrifugation, and the supernatant was removed. Granulosa cell isolation was done within 1h after follicle fluid collection to avoid post-aspiration cell death. Once isolated, the granulosa cells were divided and were either used for subsequent experiments such as RNA or protein extraction, or cultured for immunofluorescence staining, or stored at −80° C until further analysis.

### Cell culture

The KGN cells maintain the function of steroid hormone synthesis and the characteristics of granulosa cells, and are often used to study the proliferation, apoptosis, hormone secretion and receptor expression of granulosa cells. The DMEM/F12 medium was used for cell culture. The culture medium was also supplemented with 10% FBS and 100 U/mL penicillin-streptomycin (Invitrogen), as mentioned in our previous study [6]. Cells were incubated in a humidified incubator at 37° C with 5% CO_2_.

### Quantitative real-time PCR (qRT-PCR)

TRIzol reagent (Takara) was used for RNA extraction. Total RNA was quantified with a NanoDrop 2000 spectrophotometer and transcribed to cDNA with the PrimeScript RT Master Mix (RR036A, TAKARA) following the manufacturer's instructions. TB Green Premix Ex Taq II (TAKARA) was used in quantitative real-time polymerase chain reaction. Detailed primer information can be found in Supplementary Table 1.

### Western blot

Cells were lysed on ice for 30 min using ice-cold RIPA lysis buffer with protease inhibitor mix and phosphatase inhibitors. Adequate amounts of proteins were loaded into the wells of the SDS-PAGE gel. After running the gel, the proteins were transferred to a membrane (Sigma-Aldrich), which was blocked with 5% BSA for 1 hour at room temperature. The membrane was incubated with primary antibodies over night at 4° C. β-ACTIN was used as standard control. Then the membrane was incubated with secondary antibodies conjugated with HRP (Cell Signaling) at room temperature for 1 hour. Detailed information about antibodies (LNK (Santa Cruz), FOXO3 (Cell signaling, CST), phosphorylated-FOXO3 (CST), AKT (CST), phosphorylated-AKT (CST) and β-ACTIN (CST)) can be found in Supplementary Table 2.

### Expression vectors and transfection

Human LNK cDNA and mutant LNK (exon 1, PH domain, allele C was mutated to T) were cloned into respective pcDNA 3.1 vector. siRNA for LNK was synthesized. Cells were grown in 6-well plates. Transfection was performed on KGN cells using 1.0 μg/mL LNK plasmid (pc LNK), LNK mutant plasmid (mut LNK), LNK siRNA (si LNK), empty pcDNA3.1 vector (vec) or negative control RNA (neg) with Lipofectamine 2000 reagent (Thermo Fisher Scientific) following the manufacturer’s protocols. The efficiency of transfection is shown in Supplementary Figure 1A, 1B.

### Treatment with insulin

48 hours after transfection, after serum starvation for 12 h, the cells were treated with 100nM recombinant human insulin (Sigma-Aldrich) for 30 min, then they were harvested for subsequent experiments such as protein extraction.

### Annexin V-FITC apoptosis assay

Flow cytometry was performed for the assessment of apoptosis level. An annexin V-FITC/PI Double-staining Apoptosis Detection Kit (BD Biosciences) was used to stained KGN or GCs. Flow cytometry was conducted with a flow cytometer (BD FACSJazz) according to the manufacturer's protocols. Granulosa cells or KGN cells without staining were used as controls. Representative plots showing cell gating events can be found in Supplementary Figure 1C.

### TUNEL analysis

The In Situ Cell Death Detection Kit (Roche) was used for TUNEL analysis following the manufacturer’s protocols. Negative control was set following the manufacturer’s protocols: Lable Solution (without terminal transferase) was used for cell incubation instead of TUNEL reaction mixture. A confocal microscope (Zeiss LSM700) was used for observation and photography.

### Immunofluorescence

After rinsing the cells, 4% paraformaldehyde was used to fix the cells for 15 minutes at room temperature. After washing the cells, 0.1% Triton X-100 was used for permeabilization for 2 minutes on ice. 5% Bull Serum Albumin (BSA) was used for blocking. Immunofluorescence staining was performed with purified primary antibodies FOXO3a (Cell Signaling) and p-FOXO3a (Cell Signaling). Primary antibodies were diluted 1:500 and incubated at 4° C overnight. Then cells were incubated with conjugated anti-mouse or anti-rabbit secondary antibodies (CST #4408 and #8889). After washing, DAPI was added for 10 s. Cells without immunofluorescence staining were used as negative control. Detailed information about antibodies can be found in Supplementary Table 2.

### Animals

The ethics committee for animal research in Sun Yat-sen University approved the experiments and we followed the NIH Guide for the Care and Use of Laboratory Animals [30]. Mice were kept under standard conditions (12 hours light/dark, with free access to feed and water).

Whole body *LNK*-knockout (KO) C57BL/6 mice were designed by Can-ZhaoLiu, and were produced with CRISPR/Cas by Cyagen Biosciences Inc, as described in our previous study [30]. The animals were divided into four groups. Wildtype (WT) C57BL/6 mice and KO mice with normal chow were WT control group and KO control group, respectively. WT and KO mice fed with a high-fat diet and injected with dehydroepiandrosterone (DHEA, Sigma) were WT PCOS group and KO PCOS group, respectively. Typically, prepubertal mice, aged approximately 21 days, were injected daily with DHEA (PCOS group, 6 mg/100 g body weight, diluted in 200 μL sesame oil) or sesame oil only (control group) for up to 21 days. Meanwhile, mice were fed a high-fat diet (PCOS group, 60% fat calories) or regular chow (control group, 10% fat calories). The high-fat diet was also used in our previous study [30].

### Glucose tolerance test (GTT) and insulin tolerance test (ITT)

In GTT, mice were fasted for 12 hours and injected intraperitoneally with 1 g/kg body weight dextrose (Sigma). In ITT, after fasting for 6 hours, intraperitoneal 0.75 units/kg body weight insulin (Sigma) injection was performed. Glucose levels were detected from tail venous blood with an automated glucometer (Roche) at 0 min, 15 min, 30 min, 60 min, 90 min, 120 min after injection, which was also described in our previous study [30].

### Evaluation of estrus cyclicity

Vaginal cell smears were obtained from mice with normal saline at 10AM every morning for 2 weeks. They were placed on glass slides for air dry. Crystal violet was used for staining.

### Body fat assessment

EchoMRI 100 (Echo Medical Systems, US) was used to measure mice body fat rate without anesthetization. 3 repeated measurements lasted about 90 seconds were performed for each mouse. Values of fat rate were instantly generated once measurements were done and the average fat rate for each mouse was calculated.

### Tissue collection

8-week-old mice were sacrificed to collect ovarian tissue. Tissues underwent subsequent experiments such as RNA extraction or were frozen in liquid nitrogen for future analysis.

### Statistical analysis

All data were shown as mean ± SD or mean ± SEM. After normal distribution of the data was confirmed, unpaired two-tailed Student *t* test or ANOVA with Bonferroni *post hoc* test was used to test for differences. Pearson test was used to analyze correlation. SPSS 22.0 (SPSS, USA) was used for statistical analysis. Figures were generated by PRISM 7.0 (GraphPad). Statistical significance was considered to be *p*< 0.05.

### Data availability statement

The data underlying this article cannot be shared publicly due to the privacy of individuals that participated in the study. The data will be shared on reasonable request to the corresponding author.

## Supplementary Material

Supplementary Figures

Supplementary Tables
