# Supplementary material for: LNK promotes granulosa cell apoptosis in PCOS via negatively regulating insulin-stimulated AKT-FOXO3 pathway
Source: Aging (Albany NY). 2021 Jan 20;13(3):4617–33. doi: 10.18632/aging.202421 (PMC7906173; doi:10.18632/aging.202421)
Supplement: Supplementary Figures [file aging-13-202421-s001.pdf]

SUPPLEMENTARY FIGURES

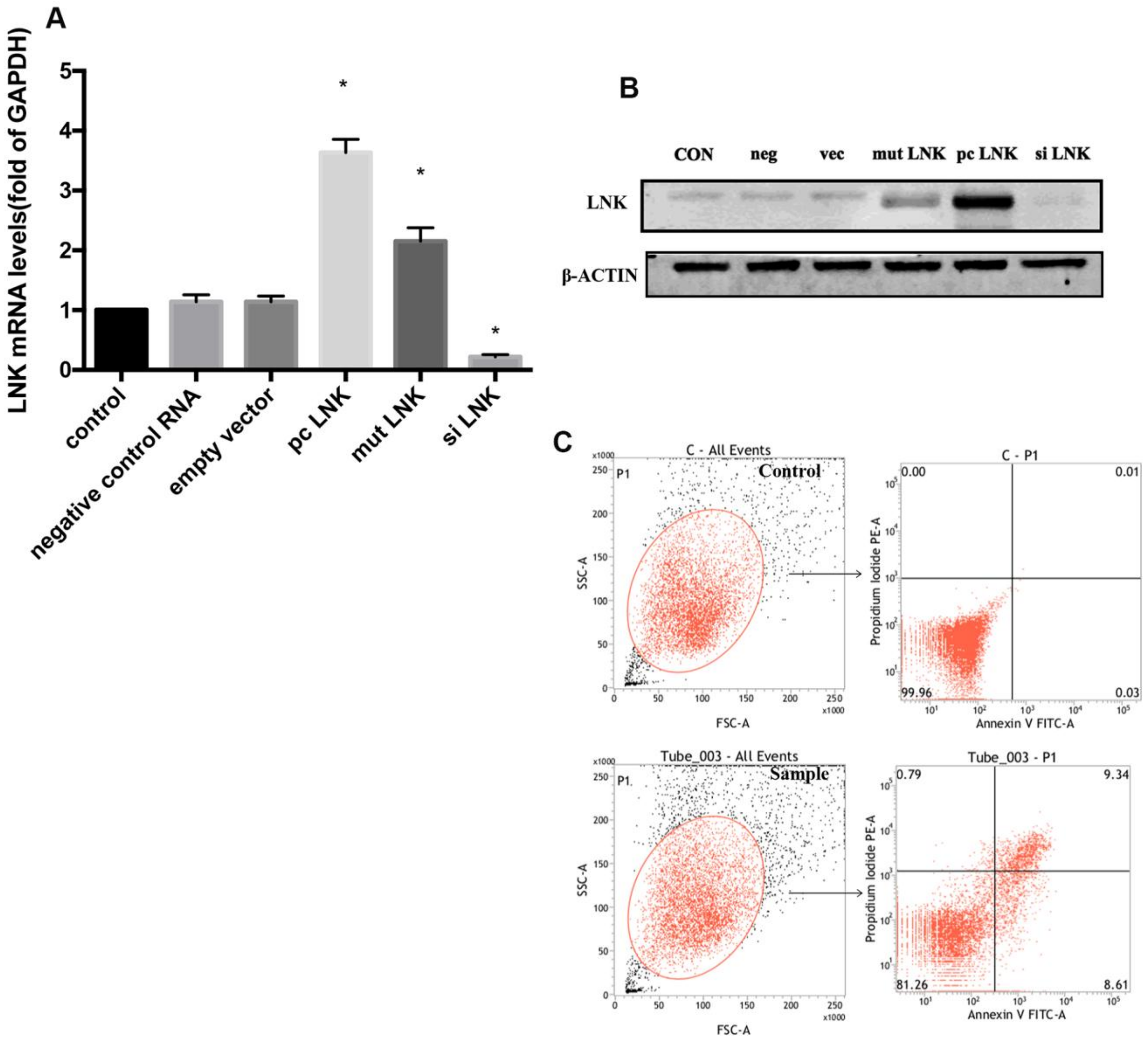

Supplementary Figure 1. (**A**, **B**) Transfection efficiency of LNK pcDNA and mutant plasmid or siRNA. The KGN cells were treated with empty culture medium (con) or transfected with LNK siRNA (si LNK) or overexpression plasmid (pc LNK) or mutant plasmid (mut LNK) or the corresponding negative control (negative control RNA (neg) or empty vector (vec)) for 48 hours. (**A**) mRN A level of LNK measured by RT-PCR. (**B**) Protein level of LNK measured by Western blot. The values were shown as means ± SD, n=3, *P<0.05 vs. Con. (**C**) Representative plots showing cell gating events of flow cytometry analysis.

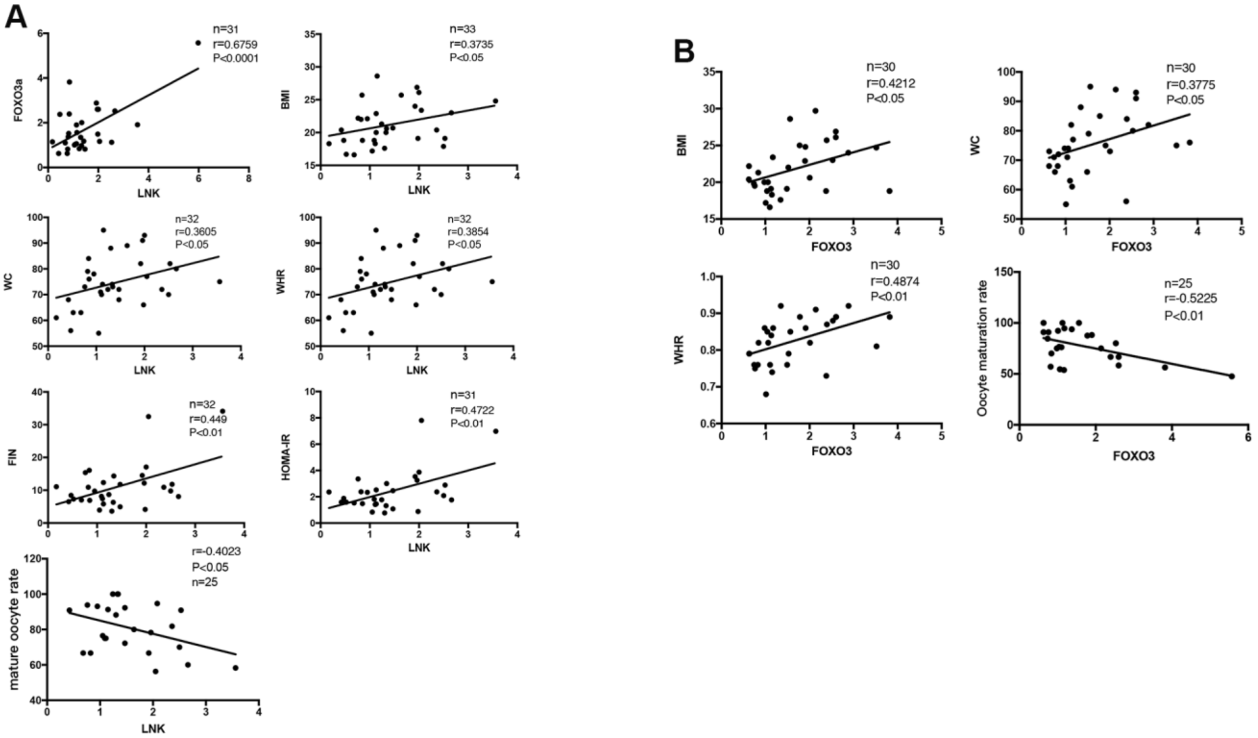

Supplementary Figure 2. (**A**) Pearson correlation analysis showed a significantly positive correlation between Lnk and FOXO3 mRNA expression in PCOS patients. Lnk mRNA level was also found to be positively correlated with clinical insulin resistance parameters in PCOS patients. A negative correlation between the level of Lnk and oocyte maturation rate in PCOS patients was also found. (**B**) FOXO3 mRNA level was found to be positively correlated with clinical insulin resistance parameters in PCOS patients. A negative correlation between the level of FOXO3 and oocyte maturation rate in PCOS patients was also shown.
