# Supplementary material for: LNK promotes granulosa cell apoptosis in PCOS via negatively regulating insulin-stimulated AKT-FOXO3 pathway
Source: Aging (Albany NY). 2021 Jan 20;13(3):4617–33. doi: 10.18632/aging.202421 (PMC7906173; doi:10.18632/aging.202421)
Supplement: Supplementary Tables [file aging-13-202421-s002.pdf]

SUPPLEMENTARY TABLES

Supplementary Table 1. Primer sequences used in quantitative RT-PCR.

Gene	Forward primer	Reverse primer
FOXO3-mouse	ACTGAGGAAAGGGGAAATGG	CAAAGGTGTCAAGCTGTAAACG
GLUT4-mouse	ACACTGGTCCTAGCTGTATTCT	CCAGCCACGTTGCATTGTA
GLUT4-human	ATCCTTGGACGATTCCTCATTGG	CAGGTGAGTGGGAGCAATCT
LNK-mouse	CCAATGTGGCGATAGAGAAAAGGTAAAG	CAAACCTCAGGTCAGCCTCTACATAGC
LNK-human	AATAGGAGGGTGGCAAGAACAG	TCCCCTAGCCCCAGTTAATTTAA
FOXO3-human	TGTTGGTTTGAACGTGGGGA	GTTTGAGGGTCTGCTTTGCC
GAPDH-human	GAGCCAAAAGGGTCATCATCTC	GGTCATGAGTCCTTCCACGATAC
FSHR-human	GCG GAA CCC CAA CAT CGT GTC	TGA AGA AAT CTC TGC GAA AGT
GAPDH-mouse	AAGCCCATCACCATCTTCCA	CCTGCCTCACCACCTTCTTG
FSHR-mouse	TGCTCTAACAGGGTCTTCCTC	TCTCAGTTCAATGGCGTTCCG
LHR-mouse	GCCGCTGCACATTTTCAAC	CAGTGGCAGAGTAGCTGCAC
AMHR-mouse	GGGGCTTTGGACACTGCTT	GTCTCGGCATCCTTGCATCTC
CYP19-mouse	AACCCCATGCAGTATAATGTCAC	AGGACCTGGTATTGAAGACGAG
AR-mouse	TCCAAGACCTATCGAGGAGCG	GTGGGCTTGAGGAGAACCAT

FOXO3, forkhead box class O3; GLUT4, glucose transporter 4; FSHR, follicle stimulating hormone receptor; LHR, luteinizing hormone receptor; AMHR, anti-Mullerian hormone type 2 receptor; CYP19, cytochrome P450, family 19; AR, androgen receptor.

Supplementary Table 2. Antibodies used in western blot(WB) and immunofluorescence(IF).

Protein	Company	Product code	Dilution	Application	Molecular weight(k Da)
Akt (pan) (11E7) Rabbit mAb	Cell Signaling Technology	4685	1:1000	WB	55-70
Phospho-Akt (Ser473) (D9E) XP® Rabbit mAb	Cell Signaling Technology	4060	1:1000	WB	55-70
FoxO3a (75D8) Rabbit mAb	Cell Signaling Technology	99199	1:1000 1:500	WB IF	70-100
β-Actin (13E5) Rabbit mAb	Cell Signaling Technology	4970	1:1000	WB	35-55
Phospho-FoxO3a (Ser253) Antibody	Cell Signaling Technology	9466S	1:1000 1:500	WB IF	70-100
LNK antibody(A-12)	Santa Cruz Biotechnology	sc-393709	1:1000	WB	55-70

Supplementary Table 3. Correlation between LNK mRNA expression in granulosa cells and clinical features in patients.

		FOXO3	BMI	WC	WHR	FPG	FIN	HOMA-I R	Oocyte maturation rate
Total population	r	0.6336	0.3648	0.3369	0.3170	-0.0530	0.3400	0.3239	-0.3519
	p	<0.0001	0.0038	0.0324	0.0460	0.6904	0.0158	0.0112	0.0353
	n	52	54	54	54	50	50	50	36
PCOS	r	0.6759	0.3735	0.3605	0.3854	0.0121	0.4490	0.4722	-0.4023
	p	<0.0001	0.0323	0.0427	0.0294	0.5430	0.0099	0.0073	0.0462
	n	31	33	32	32	31	32	31	25
control	r	0.2335	0.3328	0.1765	0.1501	0.1720	0.0037	0.0175	-0.3639
	p	0.3084	0.0835	0.3689	0.4458	0.4007	0.9858	0.9337	0.3013
	n	21	21	22	22	19	18	19	11

BMI, body mass index; WC, waist circumference; WHR, waist-hip ratio; FPG, fasting plasma glucose; FIN, fasting insulin; HOMA-IR, homeostasis model assessment for insulin resistance.

Supplementary Table 4. Correlation between FOXO3 mRNA expression in granulosa cells and clinical features in patients.

		LNK	BMI	WC	WHR	FPG	FIN	HOMA-I R	Oocyte maturation rate
Total population	r	0.6336	0.3989	0.3714	0.4166	0.2529	0.1244	0.1884	-0.4900
	p	<0.0001	0.0012	0.0220	0.0011	0.0676	0.3749	0.1767	0.0024
	n	52	47	47	47	47	47	47	36
PCOS	r	0.6759	0.4212	0.3775	0.4874	0.2075	0.0177	0.0873	-0.5225
	p	<0.0001	0.0205	0.0397	0.0063	0.2626	0.9247	0.6407	0.0074
	n	31	30	30	30	30	30	30	25
control	r	0.2335	0.1884	0.2954	0.1292	0.3144	0.4720	0.4757	-0.2162
	p	0.3084	0.3781	0.1611	0.5474	0.1542	0.0266	0.0253	0.5231
	n	21	17	17	17	17	17	17	11

BMI, body mass index; WC, waist circumference; WHR, waist-hip ratio; FPG, fasting plasma glucose; FIN, fasting insulin; HOMA-IR, homeostasis model assessment for insulin resistance.
